# What’s in a face? The role of facial features in ratings of dominance, threat, and stereotypicality

**DOI:** 10.1186/s41235-021-00319-9

**Published:** 2021-08-03

**Authors:** Heather Kleider-Offutt, Ashley M. Meacham, Lee Branum-Martin, Megan Capodanno

**Affiliations:** Department of Psychology, Georgia State University, Atlanta, GA 30030 USA

## Abstract

Faces judged as stereotypically Black are perceived negatively relative to less stereotypical faces. In this experiment, artificial faces were constructed to examine the effects of nose width, lip fullness, and skin reflectance, as well as to study the relations among perceived dominance, threat, and Black stereotypicality. Using a multilevel structural equation model to isolate contributions of the facial features and the participant demographics, results showed that stereotypicality was related to wide nose, darker reflectance, and to a lesser extent full lips; threat was associated with wide nose, thin lips, and low reflectance; dominance was mainly related to nose width. Facial features explained variance among faces, suggesting that face-type bias in this sample was related to specific face features rather than particular characteristics of the participant. People’s perceptions of relations across these traits may underpin some of the sociocultural disparities in treatment of certain individuals by the legal system.

## Significance statement

Faces judged as stereotypically Black (i.e., Afrocentric) are perceived negatively relative to less stereotypical faces, and this face-type bias influences a variety of real-world outcomes including employment and legal decisions. Dominance is a first-impression trait that is cued by facial structure and is associated with threat and criminality. In this experiment, we investigated whether facial features that are perceived as dominant and threatening, may be consistent with stereotypically Black features and thereby explain some of the biased treatment of people who have this face-type. Artificial faces were constructed to manipulate facial features to study the relations among perceived dominance, threat, and Black stereotypicality. People were shown faces with different combinations and variations, of facial features typically associated with stereotypicality; nose width, lip fullness, and variations in skin tone (here manipulated as reflectance; shadowing and texture). After presentation, people judged how well each face represented the three factors of interest (traits). Results showed that stereotypicality was related to wide nose and darker reflectance and to a lesser extent full lips; threat was associated with wide nose, thin lips, and low reflectance; dominance was mainly related to nose width. People were influenced by the facial features when making trait judgments, while the demographics of the perceiver (race, age, gender), did not change how the faces were judged. These results suggest that the extent to which people perceive dominance, threat, and stereotypicality as related, may underpin some of the sociocultural disparities in treatment of certain individuals in an applied context.

The “Barbie Bandits”, two attractive teenage girls who robbed banks in Georgia (Joseph, 2009), likely were successful during their heists because they surprised bank tellers with their atypical appearance. Jeremy Meeks, the “Sexy mugshot guy” (Rayne, 2016), who was arrested for robbery and assault, gained notoriety and a modeling contract as a result of good looks, despite his criminal activity. People judge faces quickly, making first impression judgments in as little as 100 ms (Bar et al., 2006; Willis & Todorov, 2006). Speeded judgments are often biased and based on little or no information about actual behavior (Oosterhof & Todorov, 2008). Instead, people form impressions of one another and assume character traits based in part on facial structure and the extent to which facial cues support preconceived expectations for behavior (Blair et al., 2004a, 2004b; Dotsch & Todorov, 2012; Kleider-Offutt et al., 2017a, 2017b). Face judgment research finds commonalities in facial structure that lead to judgments of dominance, trustworthiness, and a variety of other trait-based assumptions (for review Oosterhof & Todorov, 2008; Zebrowitz et al., 2011,). These judgments may play a role in how people are perceived and may relate to important applied decisions, such as political elections (Todorov et al., 2005), military rank (Mazur et al., 1984; Mueller & Mazur, 1998), and court system outcomes relating to sentence severity and guilty verdicts (Blair et al., 2004a, 2004b; Kleider-Offutt et al., 2017a, 2017b; Porter et al., 2010). These face trait judgments occur for race- and gender-ambiguous faces, suggesting that susceptibility to biased assessment may be ubiquitous (Ito et al., 2011; Kaminska et al., 2020). However, in scientific research and the news media, Black faces specifically garner biased judgment (Dixon, 2017; Dixon & Azocar, 2007; Kleider-Offutt, 2019; Kleider-Offutt et al., 2017a, 2017b). The focus of the current study is to identify facial features associated with assumed behavioral traits that underpin biased judgments of Black individuals.

Black men, specifically, are vulnerable to face-type bias and assumed criminality due to associations with the *Black man criminal stereotype* (Kleider et al., 2012; Kleider-Offutt, 2019; Kleider-Offutt et al., 2018; Knuycky et al., 2014). Black men with stereotypically Black features are often judged more negatively and more criminal in real-world and laboratory settings than are their counterparts who possess more atypical features (Blair et al., 2004a, 2004b; Kleider et al., 2012). In addition, men with more stereotypical features are more likely to be misidentified (Flowe & Humphries, 2011; Kleider-Offutt et al., 2017a, 2017b) and given more punitive sentences (Eberhardt et al., 2006) than are Black men judged as possessing fewer stereotypical features in criminal cases. For example, Black men who were misidentified as the perpetrator in a crime, incarcerated, and later exonerated based on DNA evidence (i.e., factually innocent), were judged by an independent sample of people as being more stereotypically Black than were Black exonerates who were falsely incarcerated for reasons other than eyewitness identification error (Kleider-Offutt et al., 2017a, 2017b). These findings suggest a bias to associate certain face-types with negative (e.g., criminal) actions (Kleider-Offutt et al., 2017a, 2017b).

Discussions around what drives this bias suggest that stereotypically Black features may activate negative racial stereotypes that can result in associations with fear (Golkar et al., 2015; Olsson et al., 2005). A body of research is focused on identifying what aspects of a Black face lead to negative associations for White participants. Some studies find that darker skin tone is what drives the effect (Maddox & Gray, 2002). Alternatively, some research suggests that facial features and skin tone are used together (Deregowski et al., 1975; Livingston & Brewer, 2002), while others argue that they are used independently to inform these negative associations (see for a review, Hagiwara et al., 2012; Stepanova & Strube, 2009). Although this is important work that aims to better understand what features cue negative responses, these studies did not test the specific features, or combination of features, that compose a stereotypical Black face—which is the next step in understanding why some within-race faces are judged especially harshly. One study did test specific features to determine prototypicality for several race groups. Strom et al. (2012) tested how facial metrics (e.g., face width, feature size) and skin tone influenced judgments of prototypicality across Black, White, and Korean faces. Results for Black faces showed that facial metrics had the biggest influence on White perceivers’ prototypicality ratings, while skin tone was consistently impactful for Black and Korean perceivers. Black face prototypicality was not specifically identified by metrics; however, relative to White faces, Black faces were rated as having a wider nose, thicker lips, and a wider jawline (Strom et al., 2012). Aside from this study, the bulk of the research that attributes behavioral associations to Black face-types generally suggests that stereotypicality includes some combination of a wider nose, fuller lips, and darker skin (e.g., Blair, 2006; Blair et al., 2004a, 2004b). Thus, testing and identifying what features specifically define a stereotypically Black face will inform what cues associations to criminality and negative judgments.

People have stereotypes about what makes a criminal face (MacLin & Herrera, 2006; MacLin & MacLin, 2004): they have long, shaggy, dark hair; tattoos; beady eyes; pockmarks; and scars. Faces rated high in criminality may also be identified from police lineups on appearance alone (Flowe & Humphries, 2011), and such a response is associated with *Criminal face-type bias*. Similarly, participants making speeded first impression judgments of convict faces revealed that criminality was determined immediately and was related to judgments of low trustworthiness and high dominance (Klatt et al., 2016). These studies focused on Caucasian faces, but similar biases occur for Black faces (e.g., Kleider et al., 2012).

How people form these judgments so quickly is a point of discussion. One idea is that people infer personality traits from the similarity of a person’s facial features to emotional expressions (i.e., the Emotion Overgeneralization hypothesis; Zebrowitz, 2004). Emotionally neutral faces that look angry are perceived high in dominance, while neutral faces that appear happy are perceived as trustworthy. To test the influence of these traits on criminality, Flowe and Humphries (2011) had participants rate cropped faces, such that there was no clothing or background information available, of actors and inmates on criminality, anger, dominance, trustworthiness, and maturity (i.e., baby-facedness). Results showed that, regardless of face group, both male and female faces that were judged high in criminality were also judged as high in dominance and low in trustworthiness, with angry faces being perceived as the most dominant. This suggests that a possible cue to determining that a face is threatening (i.e., associated with fear) and also criminal, is the extent to which the face looks dominant. This relationship is born out of face trait models that show that the more dominant a face is perceived, the more threatening it is judged; and these impressions of threat are closely tied to criminal appearance (Funk et al., 2017).

To investigate the relationship between facial cues and trait assessments, Oosterhof and Todorov (2008) hypothesized a framework for face evaluation. They used a data-driven approach, based on principal components analysis of 2D facial images, wherein people made judgments of face traits and then determined which facial features mapped onto which traits. Through this computational modeling approach, they could model social perception of faces tied to facial structure that influenced a specific judgment, such as dominance or trustworthiness. Using this approach, they could modify the structure of new faces to increase or decrease how trustworthy or dominant they looked. These models have been examined in several studies (Oosterhof & Todorov, 2008; Todorov et al., 2013; Walker & Vetter, 2009), suggesting that spontaneous trait inferences made based on facial appearance are derived from valence and dominance. In Todorov et al. (2008, 2011, 2013) model of face evaluation, valence is a cue to whether a person should be approached or avoided, while dominance cues the likelihood of a person inflicting physical harm. Features of faces associated with happiness and anger (i.e., valence) are overgeneralized to determine whether a person is trustworthy and should be approached or avoided. Facial features that appear dominant (e.g., looking more masculine or mature) are used to evaluate physical strength. From an evolutionary standpoint, these findings suggest that these cues are adaptive for determining who to approach and who to avoid. In support of this idea, Todorov et al. (2013) found that assessments of threat derived from facial appearance are negatively associated with perceptions of trustworthiness and positively associated with perceptions of dominance. In a similar vein, Hehman et al. (2017) investigated the contribution of dominance, trustworthiness, and youthful-attractiveness on face judgments focusing on the different contributions of the perceiver and the stimuli. They found that trait-based factors representing character (e.g., dominance) are driven more by the perceiver than are factors based on appearance (e.g., attractiveness). The authors explained how cross-classified regression can estimate the amount of variance due to faces, raters, and error, and that trait impressions are derived from several sources.

What makes a face dominant, trustworthy, and threatening is well established; what is less clear is what features or combination of features, makes a face stereotypically Black, and how those features may relate to these other traits. Could it be that features that are consistently rated as dominant are consistent with features that are rated as stereotypically Black, and therefore threatening? The current study will take the next step in addressing this gap in the literature.

We plan to evaluate whether specific facial features, or combinations of features, considered stereotypically Black are also associated with dominance and threat. We hypothesize that Black stereotypicality, dominance, and threat will be positively related traits. To test this expectation, we will focus on three main aims: (1) to examine how lip width, nose width, and skin reflectance correspond to ratings of dominance, threat, and stereotypicality; (2) to examine the extent to which rater characteristics may affect face ratings; (3) to evaluate the extent to which ratings of dominance, threat, and stereotypicality are related to each other after accounting for the effects of facial features and rater demographics.

Together these results will help to determine whether some of the bias found in judgments of more versus less-stereotypically Black faces are underpinned by feature judgments that are afforded to all faces with these features. In addition, the participant sample used in this study is primarily Black women, while much of the research to date on face-type bias focuses on a White sample. Assessing trait judgments in a sample of people who are the target of the biased judgments, will aid in understanding not only the cultural implications of face-type bias but the ubiquitous nature of such judgments. Moreover, this work addresses the need for face perception research to extend beyond primarily White samples as the fluidity of face judgments maybe based on context and the racial group that one identifies with (Willadsen-Jensen & Ito, 2008).

## Methods

### Participants

Participants were 341 Georgia State University (GSU), undergraduate psychology students. All of the students participated for course credit and self-identified their age (range = 18–51 + years old; 89.2% 18–21 years old), gender (260 female, 74 male, 5 non-binary, 2 prefer not to respond), and race (126 Black, 64 White, 71 Asian, 47 Hispanic/Latinx, 1 Native American, 28 Bi/Multi-racial, 4 other). All participants provided informed consent.

### Materials

**Fig. 1:**
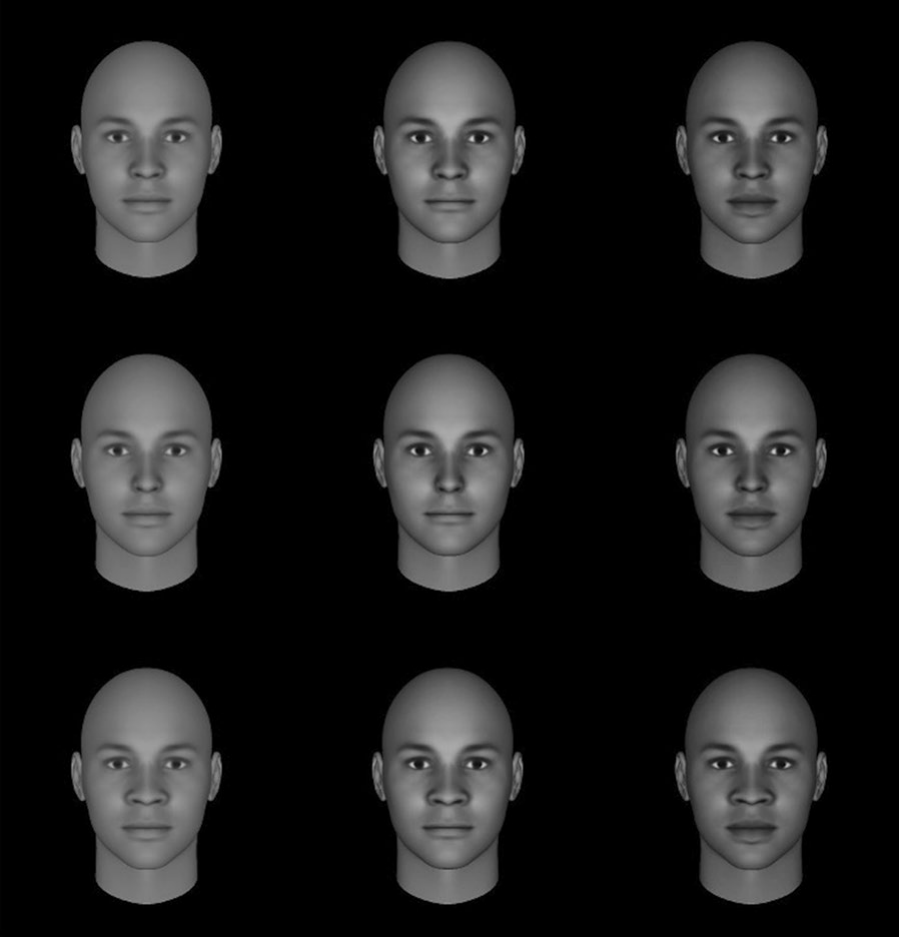
Sample stimuli. *Top row from left to right*: Baseline face (average nose and average lips); average nose and thin lips; average nose and full lips. *Middle row from left to right*: Thin nose and average lips; thin nose and thin lips; thin nose and full lips. *Bottom row from left to right*: Wide nose and average lips; wide nose and thin lips; wide nose and full lips. *Each column from left to right*: no reflectance, medium reflectance, high reflectance. Participants viewed full color images of the faces

FaceGen Modeller software (Singular Inversions, Toronto, Canada) was used to generate an average, baseline face (i.e., no feature manipulations) that was subsequently altered on different feature dimensions to create our core stimulus set. Stimuli faces were computer-generated (Fig. 1) to afford complete control over feature manipulations. Additionally, faces were presented without hair or specific skin tone (i.e., faces were racially ambiguous), such that each face was initially generated as a ‘European’ face in FaceGen Modeller and further adjusted to appear *slightly* darker in complexion utilizing the software, to isolate responses to the manipulated features as much as possible. Faces were presented in full color to participants.

Building from the average, baseline face, each successive stimulus face was manipulated to contain a specific level of nose width (wide, average, thin), lip fullness (full, average, thin), and/or reflectance (skin texture and brightness; none, medium, or high). Nose and lip features, specifically, were adjusted using the built-in sliding scale controls in FaceGen Modeller. Furthermore, each level of each feature (e.g., thin nose, full lips, etc.) was scaled to the same value for each face with that specific feature. To achieve varying levels of reflectance, we altered the contrast of the photographs (i.e., no contrast [no reflectance], 50% contrast [medium reflectance], 100% contrast [high reflectance]). It is important to note that reflectance is not meant to cue race in this paradigm, but rather we are interested in whether manipulations of skin texture and brightness, which have previously been shown to signal dominance and threat, interact with nose and lip manipulations to influence judgments of perceived stereotypically Black faces.

In total, nine faces were created with different combinations of nose width and lip fullness, and each of these nine faces was further manipulated for each level of reflectance. These three features, with three levels each, yielded a set of 27 distinct stimulus faces in total. While the stimuli set is relatively small, we have maintained maximal control over the unique faces which allowed us to assess the individual and combined influence of each feature on our outcomes of interest. Furthermore, pre-ratings of the stimuli were not collected since the goal of the study was to obtain information regarding first impressions of specifically manipulated facial characteristics (nose, lips, and reflectance).

### Procedure

In a computer laboratory with seven partitioned workspaces, each participant was randomly presented with the 27 unique facial models sequentially at the center of their computer screen. Before the presentation of each stimulus face, a fixation cross appeared in the center of the screen for 500 ms. The fixation cross was then replaced by a stimulus face for an additional 500 ms. Although prior literature has shown that individuals can form a first impression in as little as 38 ms (Oosterhof & Todorov, 2008), initial test subjects were given 100 ms to view a face. However, participants expressed stress and discomfort concerning the speed of presentation time. Thus, the stimulus presentation time was increased to 500 ms to reduce the likelihood of a potential stress response among participants, while also maintaining the desired speeded nature of the task.

Following the presentation of each face, participants provided judgments on a variety of randomized perceived inherent traits of the face and behavioral attributes (e.g., dominance, stereotypicality, threat). The full list of traits and application-based questions that were assessed, including those not used in the current report, can be found in “Appendix 1”. The response scale ranged from 1 (not at all) to 7 (extremely) for each trait judgment. Participants had unlimited time to make their response via keypress (1–7). No two participants saw the facial stimuli presented in the exact same order, nor did participants make behavioral/applied judgments in the same order for each face. Given that both the facial stimuli and associated judgments were fully randomized for each participant, we did not expect any carryover effects.

It is important to note that each of the 27 stimulus faces was shown for a total of 14 consecutive trials in which respondents would rate the face on eight traits and then respond to six applied judgment questions. These trait ratings and judgment questions are listed in “Appendix 2”. In these trials, a face would appear for 500 ms, then a judgment question, then the same face would appear for another 500 ms followed by a different judgment question, and so on until that specific stimulus had been rated on 14 different trait and judgment questions. As an initial complex multivariate model, we present in this paper an analysis of the three traits of stereotypicality, dominance, and threat.

After completing the face rating task, participants completed the Symbolic Racism 2000 Scale^1^ (Henry & Sears, 2002; not included in the following models) and a brief demographics questionnaire.

### Analysis

The model was a joint set of three multilevel regressions: responses to 27 faces within 341 raters, where each rating (dominance, threat, or stereotypicality) was predicted by facial features and rater characteristics. The general form of this model of face *f* by rater *r* can be conceptually represented by:

$$Rating_{fr} = Nose_{f} + Lip_{f} + Reflectance_{f} + Race_{r} + Age_{r} + Gender_{r} + e_{r}$$

where *Rating*_*fr*_ represents the response for that trait (stereotypicality, dominance, or threat); *Nose*_*f*_ represents the level of nose width (thin, average, or wide) for that face; *Lip*_*f*_ represents the level of lip fullness (thin, average, full) for that face; *Reflectance*_*f*_ represents skin texture and brightness (low, moderate, high) for that face, plus all two-way interactions for these three features (not shown); *Race*_*r*_ represents the race of the rater (Black, White, Hispanic/Latinx, Asian, Biracial, or other); and *e*_*r*_ is random error. The full representation of these variables and how they were coded is presented in “Appendix 2”.

This regression was fit jointly for the three traits: dominance, threat, and stereotypicality (i.e., as a simultaneous structural equation model of three rating outcomes). All models were fit in Mplus version 8.1 (Muthén & Muthén, 2017), treating the 7-point ratings as continuous (findings were highly similar when we treated the ratings as categorical, so we chose to report here the simpler, continuous score model).

## Results

The demographics of the 341 participants were modeled as dummy variables, such that White (19%), Asian (21%), Hispanic/Latinx (14%), Biracial (8%), other race (2%), non-female (male, non-binary, and prefer not to respond) participants (24%), and those older than 21 (11%) were compared to participants who identified as Black, women, and no older than 21 years old.

Because ratings were nested within faces and within raters (i.e., were cross-classified; Hehman et al., 2017), we initially fit a trivariate model of the three outcome ratings nested within faces and raters. However, in this cross-classified model, the face level was nearly fully explained, with near-zero residual variances—an understandable finding because we modeled all 27 feature patterns which were designed into the study. We therefore fit the same data to a two-level model of ratings in raters, and the model fit essentially the same (cross-classified DIC = on 96 parameters; two-level DIC = on 90 parameters). Moreover, we graphed the model-based predictions and found no strong substantive differences. We therefore present the technically simpler two-level results.

In addition, we wish to evaluate the pattern of all possible effects and to discourage the dichotomous yes/no thinking for individual effects (especially in the presence of interactions). We therefore focus on the overall model-implied effects in the graphs, presented in the appendices, which are expected to be invariant under different coding schemes. Indeed, we wish to discourage unrealistic, overly narrow reliance on p-values for individual effects because our model is attempting to capture the design of all 3 features, each with 3 levels. We therefore rely on the graphs of the model-implied effects, rather than estimates of individual parameters.

### Question 1: what features predict dominance, threat, and Black stereotypicality?

#### Effects of facial features

Facial features were modeled as contrasts of two extremes, each around an average: noses were thin, average, or wide; lips were thin, average, or full; reflectance was none, medium, or high. These features were modeled as dummy variables for thin and for wide/full (versus average) noses and lips, and for none/high versus medium reflectance (see “Appendix 2” for equations). This coding scheme allowed us to directly model every condition in the experiment, and without making assumptions of linearity or equal intervals between low, average, and high conditions of the facial features. In addition, all two-way interactions of these dummy variables were also modeled. Because these six main effects and 12 two-way interactions can be cumbersome to display and difficult to interpret, we present graphs for the model-implied effects on each of the three outcomes. The parameter estimates for the predictions by facial features are presented in “Appendix 3” Table 2.

**Fig. 2:**
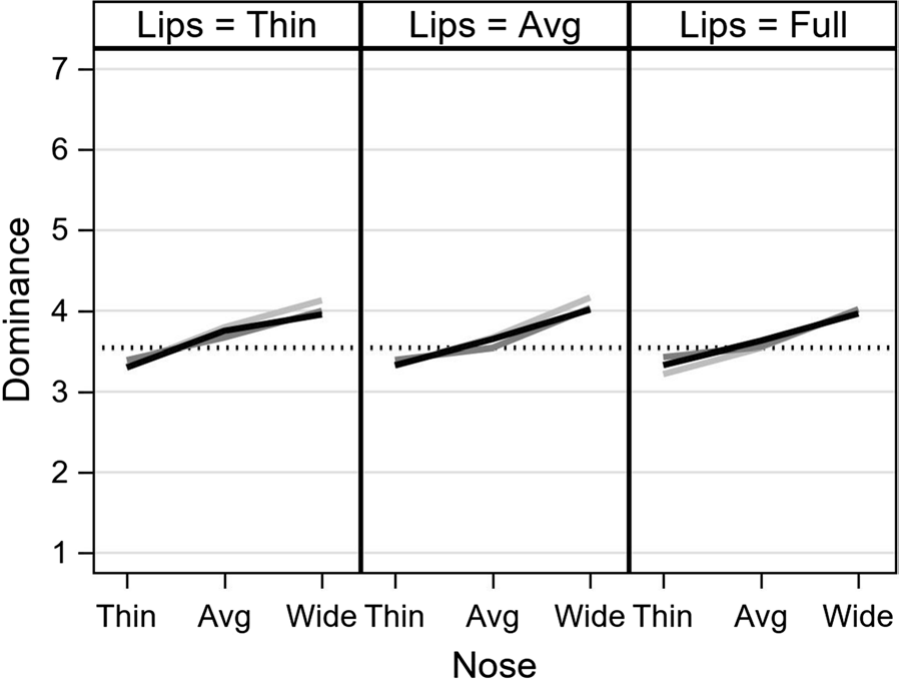
Model-predicted dominance ratings. The vertical axis is the predicted dominance score, with a dotted line for the model-implied mean. The horizontal axis represents the levels of nose width. The three lines represent degrees of reflectance (none, medium, high). Each panel represents the degree of lip fullness

Figure 2 presents the model-estimated ratings for dominance. The vertical axis is the predicted rating on the 7-point scale. The horizontal axis represents nose width: thin, average, and wide. Each panel represents one level of lip fullness: thin, average, and full. Within each panel, there is a separate line for reflectance: none (light gray), medium (gray), and high (black). The dotted horizontal line represents the model-predicted average (intercept).

The steep upward slopes in Fig. 2 show an appreciable effect for nose width, suggesting that wider noses were seen as more dominant while thinner noses were seen as less dominant. The effect of nose width ranged up to around half a unit on the 7-point scale. The other lines did not differ much from each other, and all lines in the three panels fell within half a unit of four, suggesting only small effects of lip fullness and reflectance on judgments of dominance.

**Fig. 3:**
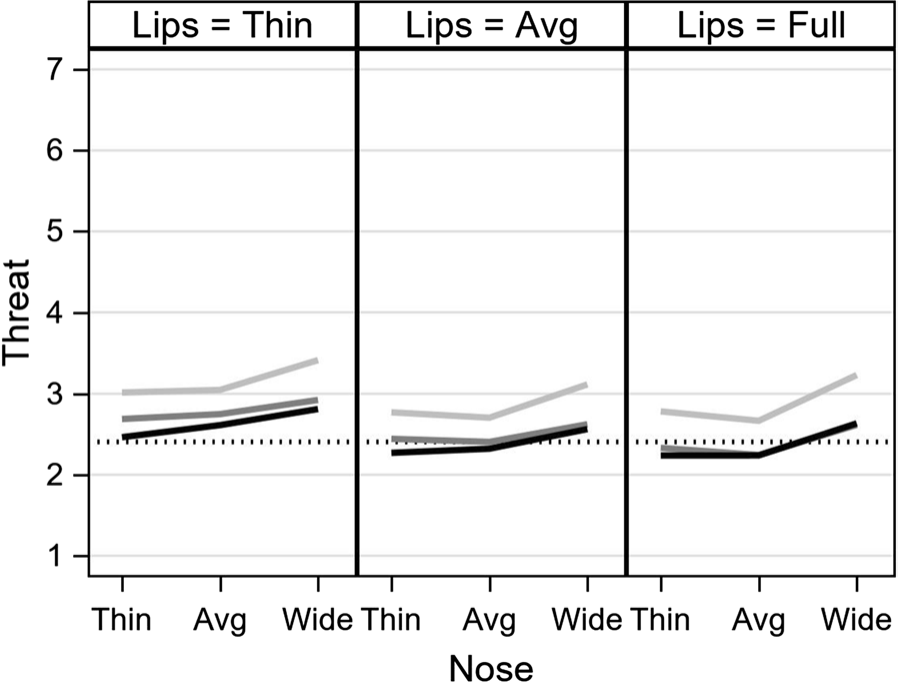
Model-predicted threat ratings. The vertical axis is the predicted threat score, with a black, dotted line for the model-implied mean. The horizontal axis represents the levels of nose width. The three lines represent degrees of reflectance (none, medium, high). Each panel represents the degree of lip fullness

Figure 3 shows the estimated ratings for threat. In all three panels, there is an upward trend for wide noses, but negligible differences for thin versus average noses. This suggests that wider noses were generally seen as more threatening. Low reflectance (light gray) is high, while there was little distinction between medium (gray) and high (black) reflectance. Thus, the absence of reflectance (i.e., none) was associated with increased judgments of threat. Thin lips (left panel) were generally more threatening than average and full lips (middle and right panels, respectively).

**Fig. 4:**
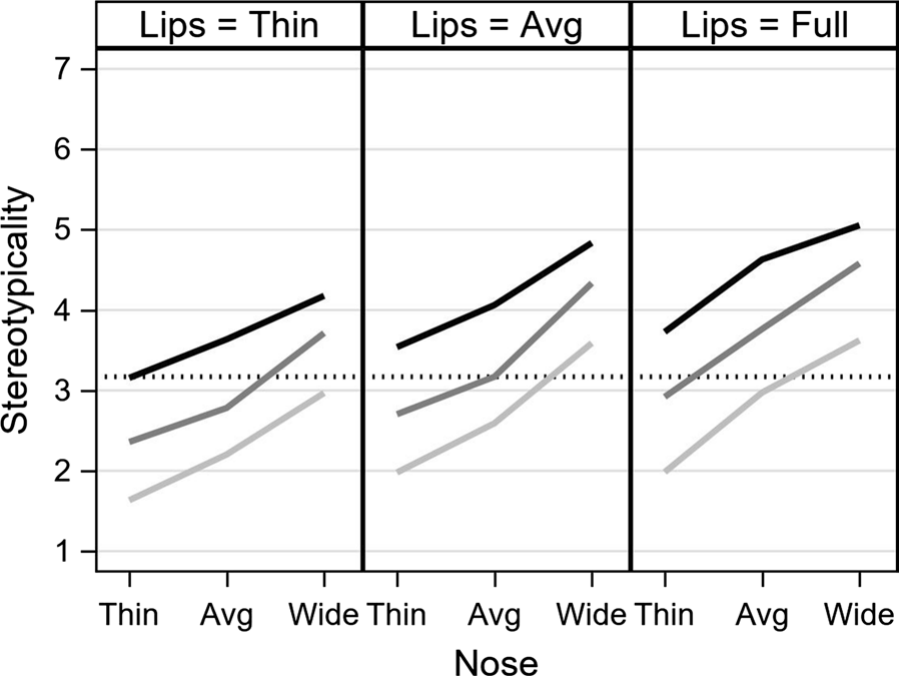
Model-predicted stereotypicality ratings. The vertical axis is the predicted stereotypicality score, with a black, dotted line for the model-implied mean. The horizontal axis represents the levels of nose width. The three lines represent degrees of reflectance (none, medium, high). Each panel represents the degree of lip fullness

Figure 4 shows the estimated total effects for stereotypicality. The steep upward slope across all three panels suggests that wider noses were seen as more stereotypical of Black faces, while thinner noses were seen as less stereotypical. In a similar fashion, there is a moderate amount of separation between high (black), medium (gray), and low (light gray) reflectance, with high reflectance positioned higher on the scale and low reflectance positioned lower. Therefore, higher reflectance was associated with increased judgments of stereotypicality (higher lines overall), whereas lower reflectance was associated with decreased judgments of stereotypicality (lower lines). Full lips (right panel) were slightly more stereotypical (higher lines) than average and thin lips (middle and left panels, respectively), although this distinction is somewhat diminished (i.e., modulated) by the combinations of nose width and reflectance.

### Question 2: do participant demographics predict judgments of dominance, threat, and Black stereotypicality?

#### Effects of rater characteristics

The parameter estimates for the rater level of the full, conditional model are presented in “Appendix 3” Table 3. The three predictors were older age (> 21 years old), race (White, Asian, Hispanic/Latinx, Biracial, or other, each coded as its own dummy variable), and non-females (91.4% male, 6.2% non-binary, 2.4% prefer not to respond). These predictors were in reference to younger adults, Black participants, and women, respectively.

Age had no substantial effects on any of the three outcomes of interest. Gender had a small effect on judgments of dominance, such that non-female participants judged faces as less dominant (β = − 0.14). Additionally, participant race had an effect on judgments of Black stereotypicality, such that White (β = − 0.31), Asian (β = − 0.21), and Hispanic/Latinx (β = − 0.20) participants judged faces as less stereotypical. Race also had a small effect on perceptions of threat, such that Asian participants rated faces as more threatening (β = 0.16).

### Question 3: what is the relationship between dominance, threat, and Black stereotypicality after controlling for the effects of participant demographics?

#### Trait correlations among raters

Table 1 shows the correlations among the three outcomes after accounting for the effects of rater demographics (i.e., rater-level random effects). The model-implied means (intercepts) and standard deviations of the ratings are shown at the bottom of the table. The three traits were all positively correlated with one another (*r* = 0.34–0.55). This finding indicates that despite rater demographics and facial features, ratings across threat, dominance, and stereotypicality were positively related.

**Table 1:** Model-based residual correlations of traits among raters

	Dom	Stereo	Threat
Dominance	1		
Black stereotypicality	.34*	1	
Threat	.55*	.34*	1
Intercept	3.54	3.17	2.41
SD	0.94	0.71	0.82

* = *p* < .01

## Discussion

Inequality in the way people are judged and ultimately treated in a variety of contexts, including the legal system, may begin with biased first impressions based on facial features. Previous research links first impression judgments of certain facial features/structure to perceptions of dominance and indicators of threat (Toscano et al., 2016). Moreover, facial features that are perceived as being stereotypically Black are touted as a harbinger for biased judgments related to criminality (e.g., Kleider et al., 2012; Kleider-Offutt et al., 2017a, 2017b). However, perceptions of faces likely result from a combination of the features of the faces as well as differences among the perceivers. The current study investigated what specific facial features were associated with judgments of Black stereotypicality and whether these features were also perceived as dominant and threatening. This research may provide initial information to better understand within-race variability in treatment and why some Black individuals are perceived as dominant and/or threatening without performing any overt actions to indicate negative behavior.

### Overview of findings

Face judgment is complex, involving faces and raters (Hehman et al., 2017). Ignoring such differences due to faces and raters may be misleading with regard to relationships across traits. For example, zero-order correlations (“Appendix 3” Table 4) would suggest that dominance is positively related to both threat (*r* = 0.34) and stereotypicality (*r* = 0.20), but that ratings of threat and stereotypicality are essentially unrelated. However, our model shows that all three traits were positively related (r = 0.34–0.55) after controlling for facial features and rater differences. Also, our results suggest that participant demographics do little to explain the relationship between these traits. This suggests that the relationship across these three traits is largely driven by facial features and not driven by the specific perceiver demographics (i.e., race, age, gender) assessed in this study. It may be that the relationships among these traits are due in part to ubiquitous facial structure cues or due to features of the perceivers not tested here.

Previous applied research on Black face-type bias describes stereotypically Black features as a combination of nose and lip width and skin tone (here reflectance); thus, this research focused on only those features. Importantly, we found that the effects of nose width, lip fullness, and reflectance had complex effects that differed by the trait being rated. A wide nose, thin lips, and the absence of reflectance were associated with higher ratings of threat. A wide nose and higher reflectance were associated with increased judgments of stereotypicality. A wide nose was the only feature substantially related to higher ratings of dominance. This suggests that a stereotypical Black face includes a wide nose and high skin reflectance but the only feature that is consistent with dominance is a wide nose. Black stereotypicality, dominance, and threat were related to faces with a wide nose. This potentially suggests that nose width is a cue indicative of a Black face but may simultaneously cue dominance and threat. Moreover, the finding that higher skin reflectance was related to Black stereotypicality but not dominance or threat, is inconsistent with other literature. If higher reflectance is a shading or texture of skin, it makes sense that this would be tied to Black stereotypicality in line with previous research (e.g., Livingston & Brewer, 2002; Maddox & Gray, 2002;). However, Todorov et al. (2013) found that in addition to dominance, threat is also cued by higher reflectance, while we found the opposite.

In line with this idea, the three traits were positively correlated among raters (*r* = 0.34–0.55), suggesting moderate to strong consistency—personal biases, not explained by demographic differences—may influence trait judgments to a fair extent. Overall, this would suggest that even after controlling for facial features and demographics, participants agree that stereotypically Black faces are dominant and threatening, to a moderate to strong degree.

Together, these results suggest that a stereotypical face-type is a combination of wide nose and higher reflectance and, to a lesser extent, full lips. Thus, a face is not likely to be judged as stereotypical based on full lips alone. This refines and validates previous work noting that a stereotypically Black face is some combination of a wide nose, full lips, and darker skin (e.g., Blair, 2006; Blair et al., 2004a, 2004b). The current study shows that among a diverse population of mostly non-White people, a stereotypical Black face is cued by a wide nose and higher reflectance. In addition, the relations among trait dominance, threat, and stereotypicality suggests that a wide nose, consistent for all three traits, may play a role in some Black people being judged as dominant and threatening. Compared to a person who is less stereotypically Black, with lighter reflectance and a relatively narrow nose, a stereotypically Black person is more likely to be judged as dominant and threatening, and potentially perceived negatively, by people making quick judgments.

This work also suggests that for people who are not White, as in our sample, Black stereotypicality is related to threat and dominance (*r* = 0.34 each). Although demographic differences did not substantially influence our outcomes, we suggest that the racial makeup of our sample may be why some of our results diverge from previous work regarding reflectance, threat, and dominance (Todorov et al., 2013). From an applied standpoint, face-type bias related to Black stereotypicality may lead to judgments of dominance, which in some circumstances is positive (e.g., boxer, military personnel), and in other circumstances less advantageous, which can lead to negative judgments. Together, these findings suggest, potentially, that when people see a stereotypically Black face, it may cue assessments of dominance and threat which are consistent traits related to criminality. Thus, it may be that some aspects of the facial features tested here underpin criminal face-type bias reported in previous research. These effects upon ratings cued by these facial features are important because without contextual information, people are left to rely on hasty first impression cues to predict traits or behavior, and perceivers are likely to rate these different traits fairly similarly.

### Limitations

While we used the demographic information available in our model, our sample of raters was primarily young, Black, females, and likely does not allow powerful tests of differences in ratings due to age, gender, and race. A more diverse sample would be informative and could yield not merely more generalizability, but interesting tests of differences in perceptions. However, it is noteworthy that our sample diverges from much of the previous research focused on face-type bias, which has tested trait assumptions within majority White samples (e.g., Blair, 2006; Blair et al., 2004a, 2004b; Eberhardt et al., 2006; Hagiwara et al., 2012). Although our study may be limited in generalizability, using a sample of people who may be the target of Black face-type bias is especially important. The findings here suggest that even for people who are part of a minoritized group and may themselves have encountered racial bias, are still prone to judge features representative of their racial group as dominant and threatening in some circumstances, lending support to the ubiquitous nature of biased racial judgments.

In addition, we intentionally used a small set of features on artificial faces. The facial features in the current study were specifically designed and controlled to test features considered to be stereotypically Black and/or dominant in previous applied studies. More variation on more features with more faces could also provide more information about effects upon perceptions.

## Conclusion

The current sociocultural climate suggests that there is a need for people to be more cognizant of how they perceive and interact with individuals from different groups. First impressions based on facial features can lead to face-type bias and can serve as a vehicle to perpetuate faulty expectations of behavior. Throughout the legal system, people are assessed from the time of first interview (e.g., when stopped on the street or pulled over in their vehicle) to trial and sentencing. An awareness of race-based biases in face judgment could be disseminated throughout the legal system as training for law enforcement and triers of fact as well as become part of jury instruction to community members who serve as jurors. An awareness of biased tendencies will not stop people from having a bias but may slow knee-jerk decisions that are made prior to considering facts and evidence. Most misidentified men who were exonerated based on DNA evidence are Black (The Innocence Project, 2021), which suggests biased expectations are at work. Knowing that some Black individuals are judged as dominant and possibly threatening based on their facial structure should encourage citizens, law enforcement, and the legal system generally, to pause before making judgments that could have long-term impact.

## Data Availability

Upon request data are available to reviewers and can be uploaded to an appropriate website.

## Appendix 1

Full list of traits assessed (1 (not at all) to 7 (extremely)):

**How ________ was the previous face?:**

- Dominant
- Threatening
- Stereotypically Black
- Stereotypically White
- Trustworthy
- Criminal-looking
- Baby-faced
- Attractive

Full list of applied questions assessed (1 (not at all likely) to 7 (extremely likely)):

**How likely is it that you would ________?:**

- Sit next to this person on the bus?
- Share an Uber with this person?
- Talk to this person at a party?
- Trust this person with your money?
- Vote for this person in an election?
- Trust this person to deliver a valuable package?

## Appendix 2

### Cross-classified model equations

The cross-classified model for *i* ratings given by *j* raters across *k* faces upon the three traits *t* was specified as:

$${\mathbf{Level}}\;{\mathbf{1}}\;\left( {{\mathbf{within}}} \right):Y_{ijkt} \, = \,{\ss}_{1jt} \, + \,{\ss}_{2kt} \, + \,e_{ijkt}$$

where

- *Y*_*ijkt*_ is the rating *i* given by person *j* for face *k* upon trait *t*.
- *ß*_*1jt*_ is the main effect of person *j* upon trait *t*.
- *ß*_*2kt*_ is the main effect of face *k* upon trait *t*.
- *e*_*ijkt*_ is random error, distributed normally with free covariance across the three traits (i.e., each with mean zero and freely estimated variance and covariances).

$${\mathbf{Level}}\;{\mathbf{j}}\;\left( {{\mathbf{raters}}} \right):{\ss}_{1jt} \, = \,\gamma_{10t} \, + \,\gamma_{11t} White_{j} \, + \,\gamma_{12t} Asian_{j} \, + \,\gamma_{13t} Hisp_{j} \, + \,\gamma_{14t} Old_{j} \, + \,\gamma_{15t} Non{ - }Female_{j} \, + \,u_{1jt}$$

where

- *γ*_*10t*_ is the grand mean of trait *t*.
- *γ*_*11t*_*White*_*j*_ is the fixed effect of White (relative to Black raters) upon trait *t*.
- *γ*_*12t*_*Asian*_*j*_ is the fixed effect of Asian (relative to Black raters) upon trait *t*.
- *γ*_*13t*_*Hisp*_*j*_ is the fixed effect of Hispanic/Latinx (relative to Black raters) upon trait *t*.
- *γ*_*14t*_*Old*_*j*_ is the fixed effect of being older than 21 years (i.e., dummy coded) upon trait *t*.
- *γ*_*15t*_*Non-Female*_*j*_ is the fixed effect of being non-female (male, non-binary, prefer not to respond) upon trait *t*.
- *u*_*1jt*_ is random error for rater *j*, distributed normally with free covariance across the three traits (i.e., each with mean zero and freely estimated variance and covariances).

$$\begin{aligned} {\mathbf{Level}}\;{\mathbf{k}}\;\left( {{\mathbf{faces}}} \right): {\ss}_{2kt} & = g_{21t} NT_{k} + g_{22t} NW_{k} + g_{23t} LT_{k} + g_{24t} LF_{k} + g_{25t} RN_{k} + g_{26t} RH_{k} \\ & \quad + g_{27t} NT_{k} * \, LT_{k} + g_{28t} NT_{k} * \, LF_{k} + g_{29t} NT_{k} * \, RN_{k} + g_{30t} NT_{k} * \, RH_{k} \\ & \quad + g_{31t} NW_{k} * \, LT_{k} + g_{32t} NW_{k} * \, LF_{k} + g_{33t} NW_{k} * \, RN_{k} + g_{34t} NW_{k} * \, RH_{k} \\ & \quad + g_{35t} LT_{k} * \, RN_{k} + g_{36t} LT_{k} * \, RH_{k} + g_{37t} LF_{k} * \, RN_{k} + g_{38t} LF_{k} * \, RH_{k} + + \, u_{2kt} \\ \end{aligned}$$

where each of the three facial features is dummy coded relative to their midpoint (zero), nose (thin, wide: NT, NW), lips (thin, full: LT, LF), and reflectance (no, high: RN, RH):

- *γ*_*21t*_&*γ*_*22t*_ are the fixed effects of thin and wide nose (relative to average nose) upon trait *t*.
- *γ*_*23t*_ &*γ*_*24t*_ are the fixed effects of thin and full lips (relative to average lips) upon trait *t*.
- *γ*_*25t*_ &*γ*_*26t*_ are the fixed effects of no and high reflectance (relative to average reflectance) upon trait *t*.
- *γ*_*27t*_ -*γ*_*38t*_ are the fixed effects of all two-way interactions of respective facial features upon trait *t*.
- *u*_*2kt*_ is random error for face *k*, distributed normally with free covariance across the three traits (i.e., each with mean zero and freely estimated variance and covariances).

### Two-level model equations

Because most of the variance components for faces (*u*_*2kt*_) in the cross-classified model estimated close to zero, we fit the model as a two-level model of *i* ratings within *j* raters for the three traits *t*. Because this is a simple restriction of the cross-classified model (no variance components for *k* faces), we keep the same notation, but drop the *k* subscripts:

$${\mathbf{Level}}\;{\mathbf{1}}\;\left( {{\mathbf{within}}} \right): Y_{ijkt} \, = \,{\ss}_{1jt} \, + \,e_{ijkt}$$

where

- *Y*_*ijt*_ is the rating *i* given by person *j* upon trait *t*.
- *ß*_*1jt*_ is the main effect of person *j* upon trait *t*.
- *e*_*ijt*_ is random error, distributed normally with free covariance across the three traits (i.e., each with mean zero and freely estimated variance and covariances).

$$\begin{aligned} {\mathbf{Level}}\;{\mathbf{2}}\;\left( {{\mathbf{raters}}} \right): {\ss}_{1jt} & = g_{10t} + g_{11t} White_{j} + g_{12t} Asian_{j} + g_{13t} Hisp_{j} + g_{14t} Old_{j} + g_{15t} Non - Female_{j} \\ & \quad + g_{21t} NT_{{}} + g_{22t} NW_{{}} + g_{23t} LT \, + g_{24t} LF \, + g_{25t} RN_{{}} + g_{26t} RH_{{}} \\ & \quad + g_{27t} NT_{{}} * \, LT \, + g_{28t} NT_{{}} * \, LF \, + g_{29t} NT_{{}} * \, RN \, + g_{30t} NT_{{}} * \, RH \, \\ & \quad + g_{31t} NW_{{}} * \, LT \, + g_{32t} NW_{{}} * \, LF \, + g_{33t} NW_{{}} * \, RN \, + g_{34t} NW_{{}} * \, RH \, \\ & \quad + g_{35t} LT_{{}} * \, RN \, + g_{36t} LT_{{}} * \, RH \, + g_{37t} LF_{{}} * \, RN \, + g_{38t} LF_{{}} * \, RH \, + \, u_{1jt} \\ \end{aligned}$$

where all parameters are the same as those provided for the cross-classified model—only the random effects for faces (*u*_*2kt*_) are excluded (i.e., restricted to zero).

## Appendix 3

See Tables 2, 3 and 4.

**Table 2:** Parameter estimates for level 1 of the two-level model

Relation/variable	Estimate	pSD	*p*	Lower	Upper	Std
*FACE STIMULI regressions*						
Dominance						
No reflectance	0.13	0.04	< .01	0.05	0.21	0.04
High reflectance	0.11	0.04	0.01	0.01	0.18	0.04
Thin nose	− 0.15	0.09	0.05	− 0.32	0.03	− 0.05
Wide nose	0.49	0.08	< .01	0.33	0.64	0.17
Thin lips	0.13	0.09	0.06	− 0.05	0.29	0.05
Full lips	0.01	0.08	0.45	− 0.15	0.17	0.01
Thin nose × thin lips	− 0.13	0.13	0.16	− 0.37	0.13	− 0.03
Thin nose × full lips	0.02	0.12	0.45	− 0.21	0.27	0.01
Thin nose × no reflectance	− 0.20	0.11	0.02	− 0.43	0.00	− 0.03
Thin nose × high reflectance	− 0.17	0.11	0.06	− 0.38	0.04	− 0.02
Wide nose × thin lips	− 0.16	0.13	0.11	− 0.41	0.09	− 0.04
Wide nose × full lips	− 0.03	0.13	0.44	− 0.24	0.27	− 0.01
Wide nose × no reflectance	0.00	0.11	0.49	− 0.27	0.21	0.00
Wide nose × high reflectance	− 0.13	0.11	0.18	− 0.33	0.10	− 0.02
Thin lips × no reflectance	0.02	0.11	0.44	− 0.17	0.23	0.00
Thin lips × high reflectance	− 0.03	0.10	0.38	− 0.23	0.19	0.00
Full lips × no reflectance	− 0.14	0.10	0.07	− 0.31	0.06	− 0.02
Full lips × high reflectance	− 0.03	0.11	0.36	− 0.24	0.17	− 0.01
Black stereotypicality						
No reflectance	− 0.58	0.06	< .01	− 0.69	− 0.47	− 0.15
High reflectance	0.89	0.05	< .01	0.78	1.00	0.23
Thin nose	− 0.47	0.12	< .01	− 0.72	− 0.28	− 0.12
Wide nose	1.17	0.11	< .01	0.95	1.36	0.31
Thin lips	− 0.39	0.11	< .01	− 0.59	− 0.16	− 0.10
Full lips	0.59	0.10	< .01	0.38	0.78	0.16
Thin nose × thin lips	0.04	0.16	0.38	− 0.24	0.36	0.01
Thin nose × full lips	− 0.38	0.16	0.01	− 0.66	− 0.03	− 0.07
Thin nose × no reflectance	− 0.15	0.14	0.18	− 0.40	0.12	− 0.02
Thin nose × high reflectance	− 0.06	0.14	0.31	− 0.34	0.22	− 0.01
Wide nose × thin lips	− 0.23	0.15	0.08	− 0.56	0.06	− 0.04
Wide nose × full lips	− 0.35	0.15	0.02	− 0.62	− 0.01	− 0.06
Wide nose × no reflectance	− 0.17	0.12	0.09	− 0.39	0.07	− 0.02
Wide nose × high reflectance	− 0.39	0.12	< .01	− 0.61	− 0.12	− 0.04
Thin lips × no reflectance	− 0.03	0.13	0.35	− 0.34	0.19	0.00
Thin lips × high reflectance	− 0.04	0.13	0.40	− 0.33	0.20	0.00
Full lips × no reflectance	− 0.21	0.13	0.05	− 0.47	0.05	− 0.02
Full lips × high reflectance	− 0.03	0.14	0.41	− 0.30	0.22	0.00
Threat						
No reflectance	0.30	0.04	< .01	0.23	.11	0.11
High reflectance	− 0.08	0.04	0.02	− 0.15	− .03	− 0.03
Thin nose	0.04	0.08	0.34	− 0.12	.01	0.01
Wide nose	0.22	0.07	< .01	0.08	.08	0.08
Thin lips	0.34	0.08	< .01	0.20	.13	0.13
Full lips	− 0.16	0.08	0.02	− 0.31	− .06	− 0.06
Thin nose × thin lips	− 0.10	0.13	0.23	− 0.33	− .02	− 0.02
Thin nose × full lips	0.05	0.12	0.34	− 0.17	.01	0.01
Thin nose × no reflectance	0.03	0.11	0.40	− 0.18	.00	0.00
Thin nose × high reflectance	− 0.09	0.11	0.22	− 0.33	− .01	− 0.01
Wide nose × thin lips	− 0.04	0.11	0.35	− 0.26	− .01	− 0.01
Wide nose × full lips	0.15	0.11	0.08	− 0.07	.04	0.04
Wide nose × no reflectance	0.19	0.10	0.02	0.01	.03	0.03
Wide nose × high reflectance	0.02	0.10	0.41	− 0.19	.00	0.00
Thin lips × no reflectance	0.04	0.11	0.39	− 0.20	.00	0.01
Thin lips × high reflectance	− 0.05	0.11	0.34	− 0.26	− .01	− 0.01
Full lips × no reflectance	0.12	0.11	0.12	− 0.09	.02	0.02
Full lips × high reflectance	0.08	0.09	0.20	− 0.09	.01	0.01
*FACE STIMULI residual variance*						
Dominance	1.72	0.03	< .01	1.66	1.77	0.96
Black stereotypicality	2.37	0.04	< .01	2.30	2.44	0.73
Threat	1.59	0.02	< .01	1.54	1.64	0.95
*FACE STIMULI covariance*						
Dominance with						
Black stereotypicality	0.25	0.02	< .01	0.22	0.30	0.13
Threat	0.38	0.02	< .01	0.34	0.42	0.23
Black stereotypicality with						
Threat	0.00	0.02	0.50	− 0.04	0.04	0.00

pSD = posterior standard deviation. p = *p *value. Lower, upper = bounds of 95% credible interval. Std = fully standardized value. Model fit DIC = 95,821 (90 free parameters). For reference, the cross-classified DIC = 95,810 (96 free parameters)

**Table 3:** Parameter estimates for level 2 (raters) of the two-level model

Relation/variable	Estimate	pSD	*p*	Lower	Upper	Std
*RATERS regressions*						
Dominance						
White	− 0.12	0.16	0.24	− 0.44	0.25	− 0.05
Asian	0.00	0.15	0.49	− 0.29	0.32	0.00
Hispanic/Latinx	0.02	0.16	0.45	− 0.31	0.37	0.01
Biracial	− 0.26	0.20	0.08	− 0.71	0.14	− 0.07
Other race	0.19	0.47	0.36	− 0.79	1.24	0.02
Non-female	− 0.34	0.13	< .01	− 0.58	− 0.08	− 0.14
> 21 years old	0.22	0.17	0.12	− 0.14	0.55	0.07
Black stereotypicality						
White	− 0.62	0.13	< .01	− 0.89	− 0.38	− 0.31
Asian	− 0.40	0.12	< .01	− 0.65	− 0.16	− 0.21
Hispanic/Latinx	− 0.45	0.14	< .01	− 0.72	− 0.16	− 0.20
Biracial	− 0.29	0.17	0.03	− 0.64	0.02	− 0.10
Other race	− 0.34	0.35	0.18	− 0.98	0.38	− 0.05
Non-female	− 0.02	0.10	0.39	− 0.22	0.18	− 0.01
> 21 years old	0.21	0.13	0.05	− 0.04	0.47	0.09
Threat						
White	0.13	0.14	0.18	− 0.15	0.39	0.06
Asian	0.34	0.13	0.01	0.06	0.58	0.16
Hispanic/Latinx	0.11	0.16	0.22	− 0.18	0.43	0.04
Biracial	− 0.16	0.18	0.17	− 0.56	0.15	− 0.05
Other race	0.38	0.38	0.14	− 0.34	1.14	0.05
Non-female	− 0.03	0.11	0.40	− 0.24	0.21	− 0.02
> 21 years old	0.13	0.17	0.19	− 0.19	0.46	0.05
*RATERS residual variance*						
Dominance	0.93	0.08	< .01	0.79	1.11	0.95
Black stereotypicality	0.54	0.05	< .01	0.44	0.64	0.88
Threat	0.72	0.06	< .01	0.61	0.84	0.95
*RATERS covariance*						
Dominance with						
Black stereotypicality	0.23	0.05	< .01	0.15	0.36	0.34
Threat	0.44	0.06	< .01	0.33	0.56	0.55
Black stereotypicality with						
Threat	0.21	0.04	< .01	0.14	0.29	0.34
*Intercepts*						
Dominance	3.54	0.10	< .01	3.34	3.75	3.59
Black stereotypicality	3.17	0.10	< .01	2.98	3.39	4.06
Threat	2.41	0.09	< .01	2.24	2.59	2.77

pSD = posterior standard deviation. p = *p *value. Lower, upper = bounds of 95% credible interval. Std = fully standardized value. Model fit DIC = 95,821 (90 free parameters). For reference, the cross-classified DIC = 95,810 (96 free parameters)

**Table 4:** Descriptive statistics (zero-order correlations)

	Dom	Stereo	Threat
Dominance	1		
Black stereotypicality	.20*	1	
Threat	.34*	.04	1
Mean	3.64	3.16	2.76
SD	1.64	1.95	1.54

* = *p* < .01

## Footnotes

1 The Symbolic Racism 2000 Scale was collected to assess participants’ views toward Black Americans. On average, participants did not have negative views (M = 13.6) and was excluded from further analysis. The measure had good reliability (a = .71).

## References

[CR1] Bar M, Neta M, Linz H (2006). Very first impressions. Emotion.

[CR2] Blair IV (2006). The efficient use of race and Afrocentric features in inverted faces. Social Cognition.

[CR3] Blair IV, Judd CM, Chapleau KM (2004). The influence of Afrocentric facial features in criminal sentencing. Psychological Science.

[CR4] Blair IV, Judd CM, Fallman JL (2004). The Automaticity of race and Afrocentric facial features in social judgments. Journal of Personality and Social Psychology.

[CR5] Deregowski JB, Ellis HD, Shepherd JW (1975). Descriptions of White and Black faces by White and Black subjects. International Journal of Psychology.

[CR6] Dixon TL (2017). Good guys are still always in white? Positive change and continued misrepresentation of race and crime on local television news. Communication Research.

[CR7] Dixon TL, Azocar CL (2007). Priming crime and activating Blackness: Understanding the psychological impact of the overrepresentation of Blacks as lawbreakers on television news. Journal of Communication.

[CR8] Dotsch R, Todorov A (2012). Reverse correlating social face perception. Social Psychological and Personality Science.

[CR9] Eberhardt JL, Davies PG, Purdie-Vaughns VJ, Johnson SL (2006). Looking deathworthy: Perceived stereotypicality of Black defendants predicts capital-sentencing outcomes. Psychological Science.

[CR10] Flowe HD, Humphries JE (2011). An examination of criminal face bias in a random sample of police lineups. Applied Cognitive Psychology.

[CR11] Funk F, Walker M, Todorov A (2017). Modelling perceptions of criminality and remorse from faces using a data-driven computational approach. Cognition and Emotion.

[CR12] Golkar A, Björnstjerna M, Olsson A (2015). Learned fear to social out-group members are determined by ethnicity and prior exposure. Frontiers in Psychology.

[CR13] Hagiwara N, Kashy DA, Cesario J (2012). The independent effects of skin tone and facial features on Whites’ affective reactions to Blacks. Journal of Experimental Social Psychology.

[CR14] Hehman E, Sutherland CAM, Flake JK, Slepian ML (2017). The unique contributions of perceiver and target characteristics in person perception. Journal of Personality and Social Psychology.

[CR15] Henry, P. J., & Sears, D. O. (2002). The symbolic racism 2000 scale. *Political Psychology, 23*, 253–283. https://psycnet.apa.org/doi/10.1111/0162-895X.00281

[CR16] Ito, T.A., Willadsen-Jensen, E.C., Kaye, J., Park, B. (2011). Contextual variation in automatic evaluative bias to racially ambiguous faces. *Journal of Experimental Social Psychology, 47*, 818–23. https://dx.doi.org/10.1016/j.jesp.2011.02.016 10.1016/j.jesp.2011.02.016 PMC3117667 21691437

[CR17] Joseph, E. (2009, February 9). *The fall of the ‘Barbie Bandits’.* ABC News. https://abcnews.go.com/Primetime/story?id=3352813&page=1

[CR18] Kaminska OK, Magnuski M, Olszanowski M, Gola M, Brzezicka A, Winkielman P (2020). Ambiguous at the second sight: Mixed facial expressions trigger late electrophysiological responses linked to lower social impressions. Cognitive, Affective, & Behavioral Neuroscience.

[CR19] Klatt T, Maltby J, Humphries JE, Smailes HL, Ryder H, Phelps M, Flowe HD (2016). Looking bad: Inferring criminality after 100 milliseconds. Applied Psychology in Criminal Justice.

[CR20] Kleider HM, Cavrak SE, Knuycky LR (2012). Looking like a criminal: Stereotypical Black facial features promote face source memory error. Memory & Cognition.

[CR21] Kleider-Offutt HM (2019). Afraid of one afraid of all: When threat associations spread across face-types. Journal of General Psychology.

[CR22] Kleider-Offutt HM, Bond AD, Hegerty SEA (2017). Black stereotypical features: When a face type can get you in trouble. Current Directions in Psychological Science.

[CR23] Kleider-Offutt HM, Bond AD, Williams SE, Bohil CJ (2018). When a face type is perceived as threatening: Using general recognition theory to understand biased categorization of Afrocentric faces. Memory & Cognition.

[CR24] Kleider-Offutt HM, Knuycky LR, Clevinger AM, Capodanno MM (2017). Wrongful convictions and prototypical Black features: Can a face-type facilitate misidentifications?. Legal and Criminological Psychology.

[CR25] Knuycky LR, Kleider HM, Cavrak SE (2014). Line-up misidentifications: When being “prototypically Black” is perceived as criminal. Applied Cognitive Psychology.

[CR26] Livingston RW, Brewer MB (2002). What are we really priming? Cue-based versus category-based processing of facial stimuli. Journal of Personality and Social Psychology.

[CR27] MacLin MK, Herrera V (2006). The criminal stereotype. North American Journal of Psychology.

[CR28] MacLin OH, MacLin MK (2004). The effect of criminality on face attractiveness, typicality, memorability and recognition. North American Journal of Psychology.

[CR29] Maddox KB, Gray SA (2002). Cognitive representations of Black Americans: Reexploring the role of skin tone. Personality and Social Psychology Bulletin.

[CR30] Mazur A, Mazur J, Keating C (1984). Military rank attainment of a West Point class: Effects of cadets’ physical features. American Journal of Sociology.

[CR31] Mueller U, Mazur A (1998). Reproductive constraints on dominance competition in male Homo Sapiens. Evolution and Human Behavior.

[CR32] Muthén, L. K., & Muthén , B. O. (1998–2017). *Mplus user’s guide* (8th ed.). Muthén & Muthén.

[CR33] Olsson A, Ebert JP, Banaji MR, Phelps EA (2005). The role of social groups in the persistence of learned fear. Science.

[CR34] Oosterhof NN, Todorov A (2008). The functional basis of face evaluation. PNAS.

[CR35] Porter S, ten Brinke L, Gustaw C (2010). Dangerous decisions: The impact of first impressions of trustworthiness on the evaluation of legal evidence and defendant culpability. Psychology, Crime & Law.

[CR36] Rayne, N. (2016, March 9). ‘Hot Convict’ Jeremy Meeks released from prison: And he’s coming home to a modeling contract. *People*. https://people.com/crime/hot-convict-jeremy-meeks-released-from-prison/

[CR37] Stepanova EV, Strube MJ (2009). Making of a face: Role of facial physiognomy, skin tone, and color presentation mode in evaluations of racial typicality. The Journal of Social Psychology.

[CR38] Strom MA, Zebrowitz LA, Zhang S, Bronstad PM, Lee HK (2012). Skin and bones: The contribution of skin tone and facial structure to racial prototypicality ratings. PLoS ONE.

[CR300] Todorov A, Baron SC, Oosterhof NN (2008). Evaluating face trustworthiness: a model based approach. Social cognitive and affective neuroscience.

[CR301] Todorov, A. (2011). Evaluating faces on social dimensions. In: A. Todorov, S. T. Fiske, & D. A. Prentice (Eds.), *Social neuroscience: Toward understanding the underpinnings of the social mind* (pp. 54–76). Oxford University Press. 10.1093/acprof:oso/9780195316872.003.0004.

[CR39] Todorov A, Dotsch R, Porter JM, Oosterhof NN, Falvello VB (2013). Validation of data-driven computational models of social perception of faces. Emotion.

[CR40] Todorov A, Mandisodza AN, Goren A, Hall CC (2005). Inferences of competence from faces predict election outcomes. Science.

[CR42] Toscano H, Schubert TW, Dotsch R, Falvello V, Todorov A (2016). Physical strength as a cue to dominance: A data-driven approach. Personality and Social Psychology Bulletin.

[CR43] Walker M, Vetter T (2009). Portraits made to measure: Manipulating social judgments about individuals with a statistical face model. Journal of Vision.

[CR44] Willadsen-Jensen EC, Ito TA (2008). A foot in both worlds: Asian Americans’ perceptions of Asian, White, and racially ambiguous faces. Group Processes & Intergroup Relations.

[CR45] Willis J, Todorov A (2006). First impressions: Making up your mind after a 100-ms exposure to a face. Psychological Science.

[CR46] Zebrowitz LA (2004). The origin of first impressions. Journal of Cultural and Evolutionary Psychology.

[CR47] Zebrowitz LA, Wadlinger HA, Luevano VX, White BM, Xing C, Zhang Y (2011). Animal analogies in first impressions of faces. Social Cognition.

